# Postoperative mortality in hip fracture patients stratified by the Revised Cardiac Risk Index: a Swedish nationwide retrospective cohort study

**DOI:** 10.1136/tsaco-2021-000778

**Published:** 2021-07-25

**Authors:** Maximilian Peter Forssten, Ahmad Mohammad Ismail, Tomas Borg, Rebecka Ahl, Per Wretenberg, Yang Cao, Carol J Peden, Shahin Mohseni

**Affiliations:** 1 Department of Orthopedic Surgery, Orebro University Hospital, Orebro, Sweden; 2 School of Medical Sciences, Orebro University, Orebro, Sweden; 3 Department of Surgery, Karolinska University Hospital, Stockholm, Sweden; 4 Clinical Epidemiology and Biostatistics, School of Medical Sciences, Orebro University, Orebro, Sweden; 5 Department of Clinical Anesthesiology, University of Southern California Keck School of Medicine, Los Angeles, California, USA; 6 Department of Anesthesiology, University of Pennsylvania, Philadelphia, Pennsylvania, USA; 7 Division of Trauma and Emergency Surgery, Department of Surgery, Orebro University Hospital, Orebro, Sweden

**Keywords:** hip fractures, risk factor, mortality, cardiovascular system

## Abstract

**Objectives:**

The Revised Cardiac Risk Index (RCRI) is a tool that can be used to evaluate the 30-day risk of postoperative myocardial infarction, cardiac arrest and mortality. This study aims to confirm its association with postoperative mortality in patients who underwent hip fracture surgery.

**Methods:**

All adults who underwent primary emergency hip fracture surgery in Sweden between January 1, 2008 and December 31, 2017 were included in this study. The database was retrieved by cross-referencing the Swedish National Quality Register for hip fractures with the Swedish National Board of Health and Welfare registers. The outcomes of interest were the association between the RCRI score and mortality at 30 days, 90 days and 1 year postoperatively.

**Results:**

134 915 cases were included in the current study. There was a statistically significant linear trend in postoperative mortality with increasing RCRI scores at 30 days, 90 days and 1 year. An RCRI score ≥4 was associated with a 3.1 times greater risk of 30-day postoperative mortality (adjusted incidence rate ratio (IRR) 3.13, p<0.001), a 2.5 times greater risk of 90-day postoperative mortality (adjusted IRR 2.54, p<0.001) and a 2.8 times greater risk of 1-year postoperative mortality (adjusted HR 2.81, p<0.001) compared with that observed with an RCRI score of 0.

**Conclusion:**

An increasing RCRI score is strongly associated with an elevated risk 30-day, 90-day and 1-year postoperative mortality after primary hip fracture surgery. The objective and easily retrievable nature of the variables included in the RCRI calculation makes it an appealing choice for risk stratification in the clinical setting.

**Levels of evidence:**

Level III.

## Introduction

The Revised Cardiac Risk Index (RCRI), originally developed by Lee *et al*, is used to evaluate the 30-day risk of postoperative myocardial infarction, cardiac arrest and all-cause mortality.^1 2^ The association of the RCRI with postoperative adverse overall outcome has been thoroughly investigated in elective general and vascular surgery, demonstrating a relationship between higher RCRI scores and a higher risk for postoperative mortality, but has not to date been extensively investigated in hip fracture patients.^2–5^

Hip fracture surgery is associated with a high risk of death. Postoperative mortality rates range from 6% to >10% at 30 days, approximately 16% at 90 days and up to 27% at 1 year.^6 7^ These high mortality rates may be attributable to the advanced age and relative frailty of hip fracture patients, compared with the general population.^7–12^ Although some countries such as the UK have shown a significant reduction in mortality since the inception of a national audit programs and increased focus on this patient group, this has not been the case worldwide.^6 7 13 14^ A multitude of interventions during the past decade such as surgical innovation, fast-track programs and management by multidisciplinary teams, have not changed outcomes significantly in many countries.^7 13 14^ With an aging global population, and the high incidence of hip fractures in the high-income world, the importance of finding a solution to the morbidity and mortality associated with hip fractures continues to increase.^15 16^ Without any further significant interventions targeted toward reducing postoperative morbidity and mortality, hip fractures will continue to represent a high economic burden for both healthcare systems and the public that funds them.^17–19^

Tools that can be used for estimating the risk of postoperative mortality can play a vital role in tailoring preoperative and postoperative care, surgical approaches, allocation of resources as well as promoting meaningful conversations with patients and their relatives when discussing expectations and treatment plans.^20^ Although a number of tools for risk assessment of hip fracture patients already exist, the RCRI has some potential advantages over existing tools in that it requires only six variables, all of which can be gleaned from the patient’s history or records. This study aimed to investigate the relationship between the RCRI score and postoperative mortality at 30 days, 90 days and 1 year in patients undergoing primary surgery for traumatic hip fractures using a large national database. The authors hypothesized that both 30-day and 90-day, as well as 1-year postoperative mortality increases with an increasing RCRI score.

## Methods

The principles of the Declaration of Helsinki and Strengthening the Reporting of Observational Studies in Epidemiology guidelines were adhered to while conducting this study.^21^ The cohorts were obtained from Rikshöft, a Swedish National Quality Register for hip fractures composed of prospectively collected data.^22^ All adults who underwent primary emergency hip fracture surgery in Sweden between January 1, 2008 and December 31, 2017 were considered for inclusion in the current study. Cases where the hip fractures were pathological or conservatively managed were not included in the original data retrieval. The data from Rikshöft were used in order to determine the date of hospital admission, age, sex, fracture type, American Society of Anesthesiologist (ASA) classification, surgical method, date of surgery and hospital discharge date. This was cross-referenced with the Swedish National Board of Health and Welfare Cause of Death and National Patient registers using the unique patients’ social security numbers. The Cause of Death Register was used to determine the time of death and follow-up time for each patient. The National Patient Register is an administrative database that has close to full national coverage; this register was used to establish the comorbidities present in the patient at the time of surgery. The comorbidity data were used to calculate both the Charlson Comorbidity Index (CCI) and the RCRI for each patient.^1 23^

### Calculating the Revised Cardiac Risk Index

The RCRI score was calculated using the variables defined by Lindenauer *et al*, namely high-risk surgery, ischemic heart disease, congestive heart failure, cerebrovascular disease, renal insufficiency and diabetes mellitus, with each variable counting as one point if present.^5^ Hip fracture surgery is considered intermediate risk surgery by the American College of Cardiology and the American Heart Association guidelines; therefore, points for high-risk surgery were not awarded to any patient in this study.^24^

### Statistical analysis

As described by Lindenauer *et al*, patients were divided into five cohorts: RCRI 0, 1, 2, 3 and ≥4.^5^ Patient demographics and clinical characteristics were compared between the cohorts. Categorical variables were reported with percentages while continuous variables were reported as a mean±SD or median and IQR. Pearson’s χ^2^ test and Fisher’s exact test were used to determine the statistical significance of differences between categorical variables. An analysis of variance (ANOVA) was performed for normally distributed continuous variables, otherwise a Kruskal-Wallis test was used. The primary outcome of interest was 30-day postoperative mortality. The secondary outcomes of interest were 90-day and 1-year postoperative mortality.

A Poisson regression model was employed to investigate the association between the RCRI score and 30-day and 90-day postoperative mortality. A Cox proportional hazards model was used to investigate the association between the RCRI score and 1-year postoperative mortality. All analyses were performed while adjusting for age, sex, type of surgery and comorbidities that were *not* already included as part of the RCRI but were included in the CCI. Multivariate imputation by chained equations was implemented in order to compensate for missing data in sex and type of surgery; logistic regression was used for binary variables, Bayesian polytomous regression for nominal variables and a proportional odds model for ordinal variables. Results are reported as incidence rate ratios (IRR) with 95% CIs for 30-day and 90-day mortality. Results are reported as HRs with 95% CIs for 1-year mortality. A Kaplan-Meier plot was generated to visualize patient survival during the first postoperative year. In order to verify the statistical significance of the linear trend of IRRs/HRs observed with an increasing RCRI score, the analyses were repeated for each outcome with the RCRI score being analyzed as a continuous variable instead of a categorical variable. The two models produced for each outcome were compared using the Bayesian Information Criterion (BIC) and the likelihood ratio (LR) test; a model with a smaller BIC score was considered better.

Statistical significance was defined as a two-sided p value <0.05. Analyses were performed using the statistical programming language R (R Foundation for Statistical Computing, Vienna, Austria)^25^ and Stata V.16.1 (StataCorp, College Station, Texas, USA).

## Results

A total of 134 915 cases met the study inclusion criteria. From the 142 171 cases originally extracted from the Rikshöft register, 2877 (2.0%) cases were excluded due to incorrectly registered data, 4358 (3.1%) were excluded since they were re-operations and 21 (0.01%) were excluded due to the patient being under 18 years of age. As the RCRI score increased the proportion of males increased (RCRI 0: 27.9% vs RCRI ≥4: 56.7%, p<0.001). There was no clinically significant difference in age between the cohorts despite the statistically significant p value (RCRI 0: 81±11 years vs RCRI ≥4: 82±8 years, p<0.001). Patients with higher RCRI scores had more comorbidities (RCRI ≥4, CCI ≥7: 99.4% vs RCRI 0, CCI ≥7: 4.9%, p<0.001) and were less fit for surgery based on their American Society of Anesthesiologists (ASA) classification (RCRI ≥4, ASA ≥3 94.1% vs RCRI 0, ASA ≥3 45.0%, p<0.001). No clinically significant difference in fracture types was observed between cohorts. Total hip replacements were more prevalent among patients with low RCRI scores, while fixations using pins or screws were more common in patients with higher RCRI scores (table 1). All comorbidities increased with higher RCRI scores except for dementia, connective tissue disease and metastatic carcinoma (table 2).

**Table 1:** Clinical demographics stratified by the RCRI

	RCRI 0 N=79 941	RCRI 1 N=36 848	RCRI 2 N=13 086	RCRI 3 N=3971	RCRI ≥4 N=1069	P value
Age, mean (SD)	81 (±11)	83 (±9)	84 (±8)	83 (±8)	82 (±8)	<0.001
Sex, n (%)						<0.001
Female	57 624 (72.1%)	24 167 (65.6%)	7605 (58.1%)	2054 (51.7%)	463 (43.3%)	
Male	22 306 (27.9%)	12 679 (34.4%)	5481 (41.9%)	1916 (48.2%)	606 (56.7%)	
Missing	11 (0.0%)	2 (0.0%)	0 (0.0%)	1 (0.0%)	0 (0.0%)	
ASA classification, n (%)						<0.001
1	6195 (7.7%)	361 (1.0%)	82 (0.6%)	17 (0.4%)	1 (0.1%)	
2	36 207 (45.3%)	9976 (27.1%)	1772 (13.5%)	266 (6.7%)	43 (4.0%)	
3	32 391 (40.5%)	22 481 (61.0%)	8778 (67.1%)	2586 (65.1%)	621 (58.1%)	
4	3551 (4.4%)	3344 (9.1%)	2224 (17.0%)	1038 (26.1%)	377 (35.3%)	
5	53 (0.1%)	47 (0.1%)	18 (0.1%)	10 (0.3%)	7 (0.7%)	
Missing	1544 (1.9%)	639 (1.7%)	212 (1.6%)	54 (1.4%)	20 (1.9%)	
CCI, n (%)						<0.001
≤4	54 490 (68.2%)	4882 (13.2%)	237 (1.8%)	2 (0.1%)	0 (0.0%)	
5–6	21 565 (27.0%)	23 854 (64.7%)	4642 (35.5%)	180 (4.5%)	6 (0.6%)	
≥7	3886 (4.9%)	8112 (22.0%)	8207 (62.7%)	3789 (95.4%)	1063 (99.4%)	
Fracture type, n (%)						<0.001
Non-displaced cervical (garden 1–2)	10 958 (13.7%)	4694 (12.7%)	1614 (12.3%)	458 (11.5%)	144 (13.5%)	
Displaced cervical (garden 3–4)	29 757 (37.2%)	13 676 (37.1%)	4872 (37.2%)	1473 (37.1%)	394 (36.9%)	
Basicervical	2619 (3.3%)	1216 (3.3%)	438 (3.3%)	166 (4.2%)	41 (3.8%)	
Pertrochanteric (two fragments)	15 610 (19.5%)	7503 (20.4%)	2683 (20.5%)	829 (20.9%)	234 (21.9%)	
Pertrochanteric (multiple fragments)	14 427 (18.0%)	6738 (18.3%)	2416 (18.5%)	731 (18.4%)	181 (16.9%)	
Subtrochanteric	6531 (8.2%)	3008 (8.2%)	1060 (8.1%)	314 (7.9%)	75 (7.0%)	
Missing	39 (0.0%)	13 (0.0%)	3 (0.0%)	0 (0.0%)	0 (0.0%)	
Type of surgery, n (%)						<0.001
Pins or screws	14 301 (17.9%)	6043 (16.4%)	2208 (16.9%)	686 (17.3%)	220 (20.6%)	
Pins or screws with sideplate	20 646 (25.8%)	9545 (25.9%)	3375 (25.8%)	1043 (26.3%)	293 (27.4%)	
Intramedullary rod	18 511 (23.2%)	8960 (24.3%)	3266 (25.0%)	1014 (25.5%)	241 (22.5%)	
Hemiarthroplasty	19 637 (24.6%)	9940 (27.0%)	3653 (27.9%)	1083 (27.3%)	283 (26.5%)	
Total hip replacement	6798 (8.5%)	2340 (6.4%)	575 (4.4%)	144 (3.6%)	32 (3.0%)	
Missing	48 (0.1%)	20 (0.1%)	9 (0.1%)	1 (0.0%)	0 (0.0%)	

ASA, American Society of Anesthesiologists; CCI, Charlson Comorbidity Index; RCRI, Revised Cardiac Risk Index.

**Table 2:** Preoperative comorbidities stratified by the RCRI

	RCRI 0 N=79 941 (%)	RCRI 1 N=36 848 (%)	RCRI 2 N=13 086 (%)	RCRI 3 N=3971 (%)	RCRI ≥4 N=1069 (%)	P value
Hypertension	20 394 (25.5%)	18 925 (51.4%)	8542 (65.3%)	2985 (75.2%)	910 (85.1%)	<0.001
Arrhythmia	7831 (9.8%)	9243 (25.1%)	5326 (40.7%)	1991 (50.1%)	607 (56.8%)	<0.001
Myocardial infarction	0 (0.0%)	2352 (6.4%)	3032 (23.2%)	1849 (46.6%)	830 (77.6%)	<0.001
Congestive heart failure	0 (0.0%)	9153 (24.8%)	7575 (57.9%)	3339 (84.1%)	1030 (96.4%)	<0.001
Peripheral vascular disease	1984 (2.5%)	1933 (5.2%)	1198 (9.2%)	552 (13.9%)	223 (20.9%)	<0.001
Cerebrovascular disease	0 (0.0%)	13 475 (36.6%)	6749 (51.6%)	2335 (58.8%)	823 (77.0%)	<0.001
Dementia	15 118 (18.9%)	8349 (22.7%)	2811 (21.5%)	818 (20.6%)	208 (19.5%)	<0.001
Chronic obstructive pulmonary disease	7084 (8.9%)	5020 (13.6%)	2363 (18.1%)	850 (21.4%)	260 (24.3%)	<0.001
Connective tissue disease	3303 (4.1%)	1983 (5.4%)	808 (6.2%)	324 (8.2%)	69 (6.5%)	<0.001
Peptic ulcer disease	1875 (2.3%)	1392 (3.8%)	698 (5.3%)	278 (7.0%)	85 (8.0%)	<0.001
Liver disease	640 (0.8%)	473 (1.3%)	170 (1.3%)	66 (1.7%)	21 (2.0%)	<0.001
Diabetes	0 (0.0%)	10 244 (27.8%)	6170 (47.1%)	2525 (63.6%)	917 (85.8%)	<0.001
Hemiplegia	337 (0.4%)	1474 (4.0%)	747 (5.7%)	254 (6.4%)	99 (9.3%)	<0.001
Chronic kidney disease	0 (0.0%)	1624 (4.4%)	2646 (20.2%)	1865 (47.0%)	810 (75.8%)	<0.001
Local tumor	7452 (9.3%)	4497 (12.2%)	1853 (14.2%)	585 (14.7%)	173 (16.2%)	<0.001
Metastatic carcinoma	1659 (2.1%)	877 (2.4%)	312 (2.4%)	91 (2.3%)	23 (2.2%)	0.009

RCRI, Revised Cardiac Risk Index.

**Figure 1:**
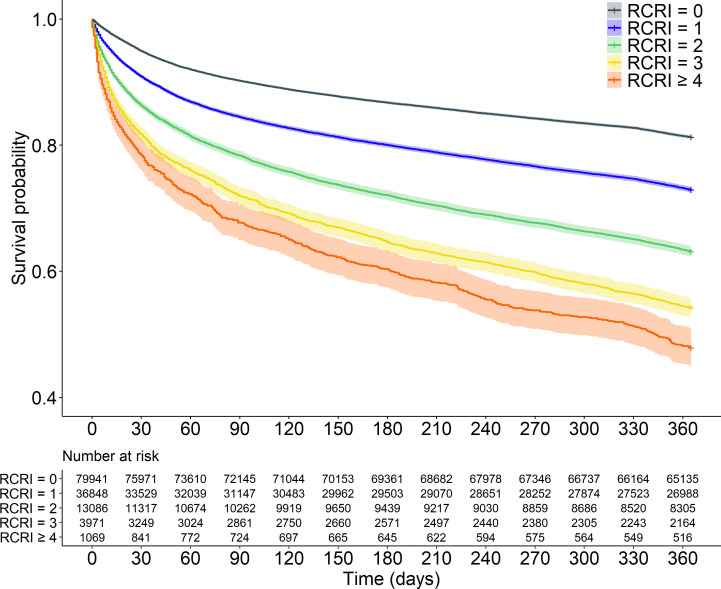
Kaplan-Meier curves describing 1-year survival for increasing Revised Cardiac Risk Index (RCRI) scores.

There was a statistically significant increase in crude postoperative mortality for each additional point on the RCRI after 30 days, 90 days and 1 year, respectively (table 3) (figure 1). In the multivariable Poisson regression analyses, statistically significant associations with increased incidences of both 30-day and 90-day postoperative mortality were found for increasing age, male sex, peripheral vascular disease, dementia, chronic obstructive pulmonary disease, liver disease, local and metastatic cancer (table 4). These same factors were found to be statistically significantly associated with an increased 1-year postoperative mortality after hip fracture surgery in the Cox regression analysis (table 5).

**Table 3:** Crude outcomes stratified by the RCRI

	RCRI 0 N=79 941	RCRI 1 N=36 848	RCRI 2 N=13 086	RCRI 3 N=3971	RCRI ≥4 N=1069	P value
Length of stay						<0.001
Median (IQR)	7 (5–11)	8 (5–12)	8 (5–13)	8 (5–13)	8 (5–13)	
Missing, n (%)	595 (0.7%)	240 (0.7%)	102 (0.8%)	36 (0.9%)	10 (0.9%)	
30-day mortality, n (%)	4077 (5.1%)	3372 (9.2%)	1802 (13.8%)	726 (18.3%)	231 (21.6%)	<0.001
90-day mortality, n (%)	7835 (9.8%)	5734 (15.6%)	2835 (21.7%)	1116 (28.1%)	345 (32.3%)	<0.001
1-year mortality, n (%)	14 465 (18.1%)	9713 (26.4%)	4734 (36.2%)	1797 (45.3%)	544 (50.9%)	<0.001

RCRI, Revised Cardiac Risk Index.

**Table 4:** Incidence rate ratio (IRR) for 30-day and 90-day mortality after hip fracture surgery

Variable	30-day IRR (95% CI)	P value*	90-day IRR (95% CI)	P value†
Revised Cardiac Risk Index (RCRI)				
0	Ref.		Ref.	
1	1.5 (1.43 to 1.58)	<0.001	1.35 (1.30 to 1.40)	<0.001
2	2.08 (1.96 to 2.21)	<0.001	1.75 (1.67 to 1.83)	<0.001
3	2.68 (2.46 to 2.91)	<0.001	2.18 (2.05 to 2.33)	<0.001
≥4	3.13 (2.72 to 3.60)	<0.001	2.54 (2.28 to 2.84)	<0.001
Age	1.08 (1.08 to 1.08)	<0.001	1.07 (1.07 to 1.07)	<0.001
Sex				
Female	Ref.		Ref.	
Male	1.92 (1.84 to 2.00)	<0.001	1.62 (1.57 to 1.67)	<0.001
Peripheral vascular disease				
No	Ref.		Ref.	
Yes	1.13 (1.03 to 1.23)	0.008	1.16 (1.09 to 1.24)	<0.001
Dementia				
No	Ref.		Ref.	
Yes	1.79 (1.71 to 1.87)	<0.001	1.77 (1.71 to 1.82)	<0.001
Chronic obstructive pulmonary disease				
No	Ref.		Ref.	
Yes	1.36 (1.28 to 1.44)	<0.001	1.27 (1.22 to 1.33)	<0.001
Connective tissue disease				
No	Ref.		Ref.	
Yes	0.97 (0.87 to 1.07)	0.509	0.97 (0.90 to 1.05)	0.438
Liver disease				
No	Ref.		Ref.	
Yes	1.88 (1.55 to 2.29)	<0.001	1.68 (1.45 to 1.95)	<0.001
Cancer				
None	Ref.		Ref.	
Local tumor	1.09 (1.02 to 1.16)	0.007	1.14 (1.09 to 1.20)	<0.001
Metastatic	1.99 (1.78 to 2.22)	<0.001	2.54 (2.36 to 2.73)	<0.001
Type of surgery				
Pins or screws	Ref.		Ref.	
Pins or screws with sideplate	1.05 (0.98 to 1.12)	0.138	1.03 (0.98 to 1.08)	0.294
Intramedullary rod	1.03 (1.97 to 1.11)	0.338	0.98 (0.93 to 1.03)	0.345
Hemiarthroplasty	1.08 (1.01 to 1.15)	0.026	0.97 (0.93 to 1.02)	0.214
Total hip replacement	0.64 (0.54 to 0.75)	<0.001	0.53 (0.47 to 0.60)	<0.001

Poisson regression model with robust SEs. Model adjusted for age, sex, comorbidities and type of surgery.

*The linear trend of the IRRs for 30-day mortality for the RCRI was tested using the LR test (p<0.001), the BIC score was reduced by 12.3 and the p value for the trend is <0.001.

†The linear trend of IRRs for 90-day mortality for RCRI was tested using the LR test (p=0.014), the BIC score was reduced by 28.3 and the p value for the trend is <0.001.

BIC, Bayesian Information Criterion; LR, likelihood ratio; Ref., reference.

**Table 5:** HRs for 1-year mortality after hip fracture surgery

Variable	HR (95% CI)	P value*
**Revised Cardiac Risk Index (RCRI**)		
0	Ref.	
1	1.32 (1.28 to 1.35)	<0.001
2	1.81 (1.75 to 1.87)	<0.001
3	2.35 (2.24 to 2.47)	<0.001
≥4	2.81 (2.58 to 3.06)	<0.001
**Age**	1.06 (1.06 to 1.06)	<0.001
**Sex**		
Female	Ref.	
Male	1.58 (1.54 to 1.62)	<0.001
**Peripheral vascular disease**		
No	Ref.	
Yes	1.27 (1.21 to 1.33)	<0.001
**Dementia**		
No	Ref.	
Yes	1.84 (1.80 to 1.88)	<0.001
**Chronic obstructive pulmonary disease**		
No	Ref.	
Yes	1.31 (1.27 to 1.36)	<0.001
**Connective tissue disease**		
No	Ref.	
Yes	1.02 (0.97 to 1.07)	0.516
**Liver disease**		
No	Ref.	
Yes	1.92 (1.73 to 2.12)	<0.001
**Cancer**		
None	Ref.	
Local tumor	1.32 (1.28 to 1.36)	<0.001
Metastatic	3.42 (3.25 to 3.6)	<0.001
**Type of surgery**		
Pins or screws	Ref.	
Pins or screws with sideplate	0.98 (0.95 to 1.02)	0.316
Intramedullary rod	0.92 (0.89 to 0.96)	<0.001
Hemiarthroplasty	0.91 (0.88 to 0.94)	<0.001
Total hip replacement	0.52 (0.48 to 0.56)	<0.001

Cox regression model adjusted for age, sex, comorbidities and type of surgery.

*The linear trend of the HRs for 1-year mortality for the RCRI was tested using the LR test (p=0.018), the BIC score was reduced by 25.3 and the p value for the trend is <0.001.

BIC, Bayesian Information Criterion; LR, likelihood ratio.

After adjusting for relevant covariates, the risk of 30-day mortality increased for every additional point on the RCRI. At an RCRI score of 1, the risk of 30-day mortality increased by 50% compared with an RCRI score of 0 (adjusted IRR 1.50, 95% CI 1.43 to 1.58, p<0.001), while an RCRI score ≥4 was associated with a mortality risk more than three times larger than that observed at an RCRI score of 0 (adjusted IRR 3.13, 95% CI 2.72 to 3.60, p<0.001) (table 4). Comparable results were observed for 90-day mortality at an RCRI score of 1 (adjusted IRR 1.35, 95% CI 1.30 to 1.40, p<0.001) and an RCRI score ≥4 (adjusted IRR 2.54, 95% CI 2.28 to 2.84, p<0.001) compared with an RCRI score of 0 (table 4). A statistically significant linear trend was found in the IRR for increasing RCRI scores for both 30-day and 90-day postoperative mortality (p<0.001). The LR tests and BIC scores support the use of RCRI scoring as a continuous variable (p<0.001 for 30-day mortality and p=0.014 for 90-day mortality) (table 4).

At an RCRI score of 1, the risk of 1-year postoperative mortality increased by 32% (adjusted HR 1.32, 95% CI 1.28 to 1.35, p<0.001), while the risk almost tripled at an RCRI score ≥4 (adjusted HR 2.81, 95% CI 2.58 to 3.06, p<0.001) (table 5). A statistically significant linear trend was found in the HR for increasing RCRI scores for 1-year postoperative mortality (p<0.001), with a smaller BIC score for RCRI as a continuous variable. The LR test was statistically significant (p=0.018) (table 5).

## Discussion

Using a large national dataset, the current study verifies previous results from our institution and improves their generalizability on the association between the RCRI and 30-day as well as 90-day postoperative mortality after isolated hip fracture surgery.^8^ A statistically significant increase in both 30-day and 90-day postoperative mortality risk was detected with an increasing RCRI score, both before and after adjusting for age, sex, type of surgery and comorbidities. This association remained up to 1 year postoperatively.

At an RCRI score of 1, the incidence of 30-day and 90-day postoperative mortality in this study was consistent with recent published national data from Denmark by Gundel *et al*, 9.2% and 15.6% vs 9.6% and 16%, respectively.^7^ The 1-year postoperative mortality has previously been reported to be between 13.4% and 27%.^7 26 27^ Comparable results were observed in the current study for 1-year mortality at an RCRI score of 0 and 1, 18.1% and 26.4%. However, when considering patients with higher RCRI scores (RCRI ≥4), the 30-day, 90-day and 1-year postoperative mortality rates were significantly higher at 21.6%, 32.3% and 50.9%. This is important to highlight in the clinical setting when discussing resource allocation between or within the hospital and orthopedic departments, or when discussing expectations of care with patients and relatives, since higher RCRI scores are associated with worse postoperative outcomes.

A number of previous studies have all found evidence of a significant increase in the postoperative 30-day risk of myocardial infarction, cardiac arrest or death with an increase in the RCRI score, which is in line with the results of the current study.^2 4 5^ However, the risks calculated by these studies were significantly lower compared with the 30-day mortality rates presented in the current study. For example, Duceppe *et al* determined the mortality rate to be 3.9% at an RCRI score of 0% and 15% at an RCRI score ≥3.^2^ These low numbers, compared with the current data presented, can be attributed to the inclusion of several other types of surgery in addition to hip fracture surgery, such as vascular and thoracic surgery, without further subgroup analyses. Hip fracture patients tend to be both older and have more comorbidities, which was also evident in our study cohort; consequently, the postoperative mortality rate would be expected to be higher since these factors are strongly associated with worse postoperative outcomes.^7–11 28–32^ Furthermore, hip fracture surgery is considered a time-dependent emergency surgery with limited time for preoperative optimization.^33^ The previous studies included both emergency and elective surgery, with the majority of cases being elective where more time for preoperative optimization is available.^2 4 5^

With the exception of some countries, such as the UK, the postoperative mortality rate after traumatic hip fracture surgery has remained static despite investments being made into monitoring outcomes, multidisciplinary management, defined standards of care and early mobilization after surgery.^6 34^ One factor which bears consideration is the finite nature of healthcare resources. In a universal healthcare system, it is not uncommon for demand to exceed the available supply. Accordingly, the proper allocation of operative resources, in the form of personnel, operating rooms and equipment, as well as access to higher levels of care, for example, intensive care, is always an ongoing discussion. This is a particularly challenging off-hours when staffing is reduced. The RCRI could potentially be used to identify which patients are in the greatest need of these limited resources; for instance, if there is a specific subgroup which would automatically benefit from admission to intensive care postoperatively or the decision to expedite or postpone surgery.

Notably, the RCRI has already been incorporated in several guidelines for cardiac protection after surgery.^24 35^ The most recent guidelines from the American College of Cardiology/American Heart Association and the European Society of Cardiology/European Society of Anesthesiology (ESC/ESA) advocate for continuing beta-blockers in patients with regular preoperative treatment.^24 35^ They also recommend considering the initiation of beta-blocker therapy in intermediate-risk to high-risk patients; these are defined as having ≥2 clinical risk factors, ASA class ≥3 and ≥3 factors from the RCRI.^24 35^ Lindenauer *et al* observed that beta-blockers had a greater protective effect after surgery in patients with a higher RCRI score.^5^ In a recent study, ongoing beta-blocker therapy was associated with reduced postoperative mortality in traumatic hip fracture patients.^12^ Further investigation is required in order to determine if the interaction observed by Lindenauer *et al* is also present in this more homogenous patient population consisting only of isolated hip fractures.

Other indices are used to predict postoperative mortality after hip fracture surgery, such as the Nottingham Hip Fracture Score (NHFS), the CCI and the Physiological and Operative Severity Score for the Enumeration of Mortality and Morbidity (POSSUM).^23 36–38^ However, compared with the RCRI, these tools have some limitations. Principally, they all require more variables than the RCRI, many of which are more complex than a binary yes-or-no answer.^23 36–38^ Both the NHFS and POSSUM also need blood tests performed in order to be calculated; POSSUM adds additional complexity through the use of vital signs and intraoperative data.^36–38^ The NHFS also requires additional calculation of a completely different score, the Abbreviated Mental Test score, as part of its model.^36^ In contrast, the RCRI is limited to six objective variables, all of which can be retrieved at the time of admission from the patient or the patient’s past medical records without additional blood tests or intraoperative data.^1^

The results from the currents study demonstrated the potential utility of the RCRI as a component in the preoperative assessment of hip fracture patients. Of further interest is the statistically significant linear trend in the IRRs and HRs observed for each additional point on the RCRI. This indicates that the RCRI should be used as a scale, rather than a binary classifier.

This study benefits from a nationwide sample population which is significantly larger compared with our institution’s previous study investigating the relationship between the RCRI and mortality 30 days and 90 days after hip fracture surgery.^8^ The Rikshöft register is used by all orthopedic departments in Sweden and has a case coverage ranging between 80% and 90%.^11^ Sweden does differ slightly compared with the rest of the world in the regard that it has one of the highest incidences of hip fractures.^15 16^ On the other hand, Sweden has the advantage of providing universal healthcare for its residents which removes many of the socioeconomic barriers that might result in certain hip fracture patients going undiagnosed and undertreated for serious health conditions. Nevertheless, these results must be interpreted in the context of this being a retrospective study.

Furthermore, while the predictive ability of the RCRI compared with other available risk scoring tools has not been explored in the current study, the authors intend to investigate this in the near future. Additional studies examining how the RCRI can be used to modify patient management are also indicated. In addition to providing an indicator for initiating beta-blocker therapy in some patients,^24 35^ it may also help identify patients where it is advantageous to delay surgery in order to improve preoperative optimization. There are several national guidelines in place for decreasing time from admission to surgery for hip fractures.^11 39 40^ Future studies may use the RCRI, or other risk stratifying tools, to determine if all patients benefit from such guidelines. Moreover, in frail geriatric patients with displaced cervical hip fractures, the RCRI could potentially be useful in determining if all patients should receive a hemiarthroplasty, or if there is a subgroup who might benefit from a less invasive operation using pins or screws.

## Conclusion

An increasing RCRI score is strongly associated with an elevated risk of 30-day, 90-day and 1-year postoperative mortality after primary hip fracture surgery. Potentially, the index can be used as a tool for detecting patients in need of focused preoperative optimization and higher levels of postoperative care in order to prevent adverse postoperative outcomes. The RCRI could also be used in monitoring quality of care and benchmarking, as well as when discussing treatment strategies with patients and their relatives.

## Data Availability

All data and codes are available for retrieval on reasonable request after additional approval by the Swedish Ethical Agency has been granted.
